# Prevalence of Hyperuricemia and the Use of Allopurinol in Older Poles—Results from a Population-Based PolSenior Study

**DOI:** 10.3390/ijerph18020387

**Published:** 2021-01-06

**Authors:** Mateusz Winder, Aleksander J. Owczarek, Małgorzata Mossakowska, Katarzyna Broczek, Tomasz Grodzicki, Łukasz Wierucki, Jerzy Chudek

**Affiliations:** 1 Department of Internal Medicine and Oncological Chemotherapy, Medical University of Silesia, 40-029 Katowice, Poland; chj@poczta.fm; 2 Department of Statistics, Department of Instrumental Analysis, Faculty of Pharmaceutical Sciences in Sosnowiec, Medical University of Silesia, 41-200 Katowice, Poland; aowczarek@paintbox.com.pl; 3 International Institute of Molecular and Cell Biology, 02-109 Warsaw, Poland; mmossakowska@iimcb.gov.pl; 4 Department of Geriatrics, Medical University of Warsaw, 02-007 Warsaw, Poland; kbroczek@gmail.com; 5 Department of Internal Medicine and Gerontology, Jagiellonian University Medical College, 31-531 Krakow, Poland; tomekg@su.krakow.pl; 6 Department of Preventive Medicine and Education, Medical University of Gdańsk, 80-210 Gdansk, Poland; wierucki@gumed.edu.pl

**Keywords:** hyperuricemia, prevalence, elderly, allopurinol

## Abstract

Background: Our study analyzes the frequency and risk factors of hyperuricemia and the use of allopurinol in a representative cohort of the older Polish adult population. Methods: The analysis was a part of a cross-sectional PolSenior study on aging in Poland. The complete medication data were available in 4873 out of 4979 community dwelling respondents aged 65 and over. Serum uric acid concentrations were evaluated in 4028 participants (80.9% of the cohort). Results: Hyperuricemia was observed in 28.2% of women and 24.7% of men. Ten risk factors of hyperuricemia were selected based on multivariable LASSO logistic regression analysis. Nine factors showed significant odds ratios: eGFR < 60 mL/min/1.73 m^2^ (OR = 4.10), hypertriglyceridemia (OR = 1.88), obesity (OR = 1.75), heart failure (1.70), CRP > 3.0 mg/dL (OR = 1.64), coronary artery disease (OR = 1.30), use of loop-diuretics (OR = 4.20), hydrochlorothiazide (OR = 2.96), and thiazide-like diuretics (OR = 2.81). Allopurinol was used by 2.8% of men and 1.8% of women. The therapy was considered effective in 46.7% of men and 53.3% of women. Conclusions: Hyperuricemia was present in 23.1% (95% CI: 21.8–24.4) of the older Polish population. The frequency of hyperuricemia increases with age, reaching 30.5% in men and 33.7% in women aged 90 years or more. Chronic kidney disease, obesity, heart failure, hypertriglyceridemia, and the use of diuretics were the strongest risk factors for hyperuricemia in older adults. The treatment with allopurinol was ineffective in more than half of participants.

## 1. Introduction

Uric acid (UA) is a product of purine nucleotides metabolism, a process catalyzed by the enzyme xanthine oxidase (XO). UA has antioxidant properties comparable to those of ascorbate [1], but higher overall concentration of UA in humans makes it one of the most important antioxidants, responsible for neutralizing more than 50% of free radicals such as reactive oxygen species (ROS) [2]. However, the excess serum uric acid (SUA) concentration has negative health effects.

About 70% of UA is excreted with urine, while the remaining 30% is eliminated via the digestive tract. Urate transporters (URAT), i.e., URAT1 and voltage-driven urate transporter 1 (URATv1) responsible for UA reabsorption in the renal tubules are also found in other tissues, including vascular endothelial cells. UA absorbed into the endothelium causes inflammation and oxidative stress by reducing the bioavailability of nitric oxide (NO) due to the decreased catalytic activity of endothelial NO synthase (eNOS) [3,4]. Consequently, the described processes lead to endothelial dysfunction and related cardiovascular complications. High SUA levels, besides causing gout, are associated with a higher prevalence of cardiovascular disease (CVD) and increased mortality [5,6,7].

It was shown that the process of purine catabolism also generates ROS, superoxides and peroxynitrite concurrently with UA, indicating that XO activity could be the target in the treatment of CVD resulting from endothelial damage [3]. The role of XO activity in the endothelial dysfunction was demonstrated in the studies of the effect of XO inhibitors on improving endothelial function in patients with metabolic syndrome, type 2 diabetes, chronic kidney disease (CKD), chronic heart failure (CHF), coronary artery disease (CAD) and in heavy smokers [8,9,10,11,12,13,14]. Moreover, XO is directly involved in the progression of atherosclerosis by activating macrophages to uptake the low-density lipoproteins (LDL) and facilitating their transformation into foam cells inside atherosclerotic plaques [15].

A widely accepted definition of hyperuricemia has not been elaborated and there is no clear laboratory reference range for SUA. In in vitro studies, crystallization of monosodium urate (MSU) at physiological pH and temperature is observed when the UA concentration exceeds 6.8 mg/dL. However, at lower pH and temperature, crystal formation may occur at the UA concentration of 6.0 mg/dL [16].

The range of physiological SUA concentration is estimated between 1.5–6.0 mg/dL in women and 2.5–7.0 mg/dL in men [17], and most authors define hyperuricemia as when SUA exceeds the values between 6–7 mg/dL. The proposed cut-off values for SUA in the general population are 6.8 or 7 mg/dL, whereas studies providing gender dependent values suggest 5.7 mg/dL [18], or often 6 mg/dL in women and 7 mg/dL in men [19]. Recommendations from epidemiology conference held in Rome in 1963 suggested these cut-off values for SUA at 7 mg/dL in men and 6 mg/dL in women [20]. A statistical approach defines hyperuricemia as an SUA concentration of more than two standard deviations above the mean [21], which makes it a standard variable for people of different ages, origins, and sexes. Furthermore, the American College of Rheumatology indicates that during urate-lowering therapy (ULT), SUA target concentration should be <6.0 mg/dL in patients with gout symptoms and <6.8 mg/dL for patients regardless of the disease activity [22,23,24].

Concerning the above, the global prevalence of hyperuricemia in the general population is difficult to estimate because it varies significantly in different countries and regions, ranging from 4.9% in Chinese women to 24.4% among the population of Okinawa, Japan, and 27.1% in the Maori population of New Zealand [18,25,26,27,28].

Main factors causing hyperuricemia are purine and fructose-rich foods (red meat, poultry, seafood, potatoes, tea, and sweet drinks), high alcohol consumption, chronic kidney disease, hypothyroidism, cachexia, myelo- and lymphoproliferative diseases, tumor lysis syndrome in oncological patients, and relatively rare genetically determined hyperuricemia [29,30,31,32,33].

Older adults are particularly prone to hyperuricemia due to the high prevalence of CKD, obesity and polytherapy. Diuretics, especially, interfere with UA excretion [32,34,35]. The antiuricosuric effect of acetylsalicylic acid is caused by the stimulation of URAT1 and occurs only in low doses [36]. Factors associated with the occurrence of hyperuricemia in older adults are declining physical activity, obesity, advanced age, sex, ethnicity, comorbidity, medications and genetic factors modulating the transport of UA in the kidneys and intestine [37]. The prevalence of hyperuricemia in older populations ranges from 7.4% in Chinese 60–69-year-olds (10.3% in >70-year-olds) [27], 20.3% in Italians over the age of 65 [38], 21% in American (USA) citizens over the age of 65 [39], 24% in the Spanish population over the age of 55 [40], and 32.5% and 32.1% in Taiwanese men and women over the age of 65, respectively [41,42]. The highest incidence of hyperuricemia was found in Ireland, reaching 27.7% in 60–80-year-olds and 43.0% in people over 80 years of age [43].

To the best of our knowledge, there are no data concerning the prevalence of hyperuricemia in Poland and other Central European countries. Increased occurrence of hyperuricemia in older adults makes this aspect important in the contexts of postulated cardiotoxicity.

In this study, we analyzed the prevalence and risk factors of hyperuricemia in the older Polish population and evaluated the use of xanthine oxidase inhibitors in equally sized five-year cohorts of men and women. The PolSenior study created the opportunity to also analyze the frequency of hyperuricemia in the oldest members of society (>90 years old).

## 2. Materials and Methods

### 2.1. Study Population

The analysis was a part of a PolSenior study on aging in Poland, performed between 2007 and 2011. This large, nationwide, multi-center, interdisciplinary project assessing social and health status of older people in Poland included 4979 subjects in 7 age cohorts (65–69 years, 70–74 years, 75–79 years, 80–84 years, 85–89 years, and 90 years or over), representative of the older Polish population, according to the protocol that has previously been described in detail [44]. All subjects signed informed consent, and assessments were carried out according the protocol approved by the Bioethics Committee of the Medical University of Silesia (KNW-6501-38/I/08).

**Figure 1:**
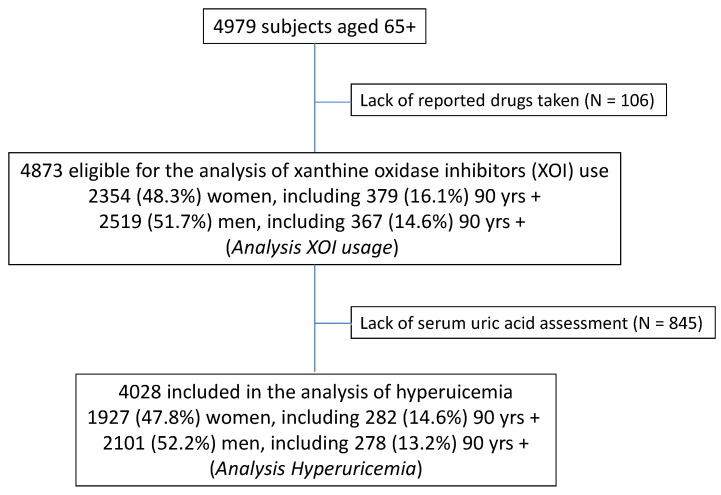
Flow chart.

For the purpose of this paper, two types of analyses were performed. The first only included individuals with serum uric acid measurements (N = 4028; 80.9% of study subjects) to assess correlates of hyperuricemia (Figure 1). The lack of SUA measurement was almost exclusively caused by the refusal of blood donation. In the second analysis, all subjects with a known medication inventory (N = 4873; 97.8% of study subjects) were included to analyze the use of xanthine oxidase inhibitors (Analysis II).

### 2.2. Biochemical Measurements

All biochemical assessments were performed in frozen samples in one laboratory selected for the purpose of the PolSenior project. Serum total cholesterol, LDL and HDL fractions, triglycerides, glucose, albumin, creatinine, high-sensitivity C-reactive protein (hsCRP), and UA concentrations were assessed by an automated system (Modular PPE, Roche Diagnostics GmbH, Mannheim, Germany) with inter-assay coefficients of variability below 1.7%, 1.2%, 1.3%, 1.8%, 1.7%, 1.7%, 2.3%, 5.7%, and 1.7%, respectively.

### 2.3. Data Analysis

Hyperuricemia was defined as SUA levels above 6 mg/dL in women and 6.8 mg/dL in men (established for population sex specific ranges corrected for the UA solubility in water [32]), or the use of xanthine oxidase inhibitors.

The treatment of hyperuricemia with xanthine oxidase inhibitors was considered to be effective if SUA was below 6 mg/dL in both sexes [22].

Nutritional status was defined based on body mass index (BMI), according to WHO criteria as: underweight (<18.5 kg/m^2^), normal weight (18.5 ÷ 24.9 kg/m^2^), overweight (25.0 ÷ 29.9 kg/m^2^), obesity (≥30.0 kg/m^2^) [45].

The waist circumference (WC) thresholds for abdominal obesity in the Caucasian population were adopted from the International Diabetes Federation—IDF (≥94 cm in men and ≥80 cm in women) [46].

Hypertension was diagnosed based on an average from four measurements performed on two separate visits, according to the 2013 ESH/ESC Guidelines for the Management of Arterial Hypertension—values equal to or higher than 140 mmHg (systolic BP) and/or 90 mmHg (diastolic BP), or antihypertensive treatment, as described previously [47].

Diabetes was defined as a fasting plasma glucose level ≥126 mg/dL or antidiabetic medications were used; hypercholesterolemia was defined as when total cholesterol levels were greater than 190 mg/dL or statins were used; and hypertriglyceridemia was defined as when serum triglyceride (TG) level was above 150 mg/dL or fibrates were used [48,49].

Glomerular filtration rate (eGFR) was estimated according to the full Modification of Diet in Renal Disease (MDRD) formula [50].

CHF and CAD were analyzed based on the participants’ reports.

### 2.4. Sociodemographic Variables

The demographic and socioeconomic status was assessed using data derived from the questionnaire (place of residence, educational level, personal income, frequency of alcohol consumption). The education level was defined as “lower” (lack of education, primary, junior high, vocational, or secondary) and “higher” (university level education). Personal income in Polish złoty (PLN) after paying taxes was voluntarily reported and evaluated against the average retirement pension in Poland in 2009 as low (<1000 PLN per month; below 100% of average), moderate (1001–2000 PLN per month—over but <200% of average), and high (>2000 PLN per month). Respondents were classified as “drinking” if they reported alcohol consumption at least three times per month.

### 2.5. Statistical Analysis

Basic statistical analyses were performed using STATISTICA 13.0 PL (TIBCO Software Inc., Palo Alto, CA, USA), while the relationship between SUA levels and age in men and women was assessed in StataSE 13.0 (StataCorp LP, TX, USA). R software, a language and environment for statistical computing, was used to assess risk factors of hyperuricemia (R Core Team (2013), R Foundation for Statistical Computing, Vienna, Austria, http://www.R-project.org/). Statistical significance was set at a *p*-value below 0.05. All tests were two-tailed. Imputations were not performed for missing data. Nominal and ordinal data were expressed as percentages. Interval data were expressed as the mean value ± standard deviation in the case of normal distribution. In the case of data with skewed or non-normal distribution, they were expressed as the median, with lower and upper quartiles. Distribution of variables was evaluated by the Anderson–Darling test and the quantile–quantile (Q–Q) plot. Homogeneity of variances was assessed by the Levene test. Comparison between two groups were done with the Student’s *t*-test for independent groups in the case of interval data. Nominal and ordinal data were compared with the χ^2^ test. The correlation coefficient between age and SUA level was calculated according to Pearson.

Risk factors of hyperuricemia were evaluated with univariable logistic regression. Based on obtained results, the LASSO logistic regression was performed to choose the best multivariable model. Then, based on the selected variables, multivariable logistic regression was performed to show corresponding odds ratios (OR) with confidence intervals (±95% CI) and *p*-values. The relationship between SUA and age was modelled with local polynomial smoothing regression with confidence intervals.

Weights specific to age, gender, and size of residence, pertinent to the 2009 population of Poland according to the Central Statistical Office (Warszawa, Poland) were used to calculated the population prevalence of hyperuricemia.

## 3. Results

### 3.1. Prevalence of Hyperuricemia

This analysis included 2101 men and 1927 women, almost half (46.4%) were aged 80 years or more and 13.9% were ≥90 (Table 1). There were 2395 (59.4%) rural dwellers. One in five participants (20.2%) lived alone. Only 57 respondents (1.4%) declared vegetarianism. Decreased eGFR (below 60 mL/min/1.73 m^2^—the cut-off point for the diagnosis of chronic kidney disease) was observed in 36.5% of the subjects. Hypertension was the most common CVD (73.4%). Diuretics were used by 29.7% participants, most frequently thiazide-like (indapamide). In addition, one-third (33.9%) were treated with aspirin.

**Figure 2:**
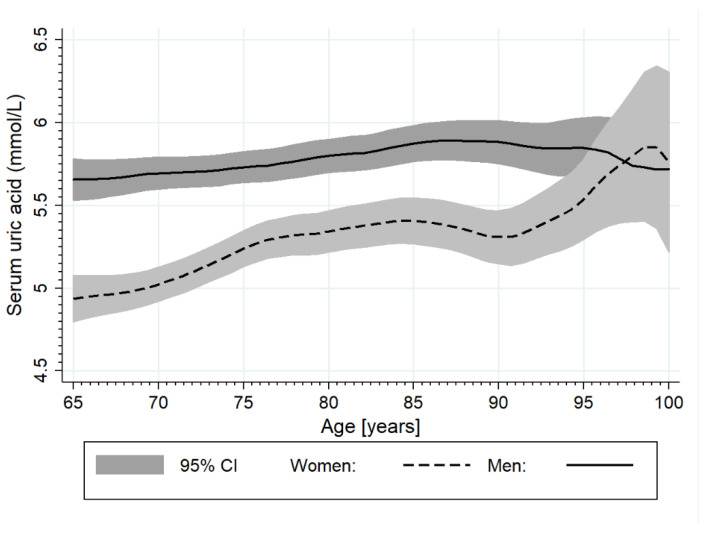
The relationship between serum uric acid (SUA) levels and age in men and women separately.

Mean SUA level was 5.5 ± 1.6 mg/dL, regardless of inclusion/exclusion of subjects based on allopurinol usage. Higher levels were observed in men than in women (5.8 ± 1.6 vs. 5.2 ± 1.5; *p* < 0.001). There was a weak positive linear correlation between age and SUA level (r = 0.118; *p* < 0.001), more pronounced in women. For each decade of life, the mean SUA level rose by 0.2 mg/dL (Figure 2).

Hyperuricemia, as defined by SUA ≥ 6 mg/dL in women and ≥ 6.8 mg/dL in men, was observed in 544 (28.2%) women and in 520 (24.7%) men (*p* < 0.05). The weighted prevalence of hyperuricemia for the Polish population was estimated at 23.1% (95% CI: 21.8–24.4), being higher among women (25.5%; 23.4–27.4) than men (19.2%; 17.5–20.9).

**Figure 3:**
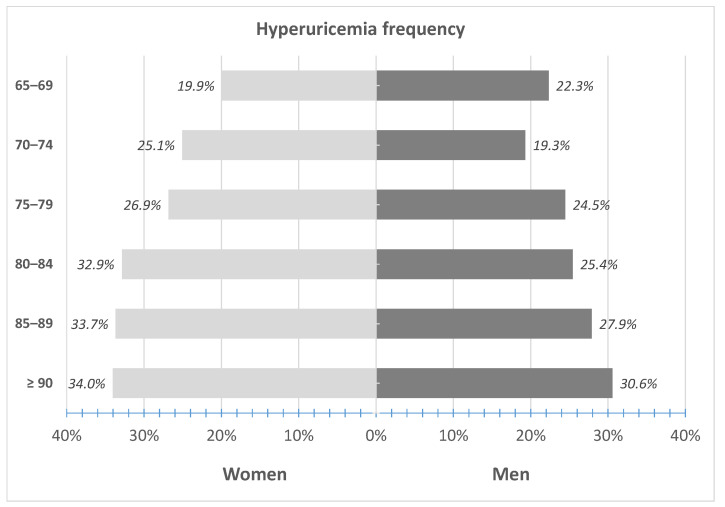
Frequency of hyperuricemia (defined by SUA ≥ 6 mg/dL in women and ≥ 6.8 mg/dL in men) in 5-year interval subgroups.

Only 62 (11.9%) men and 37 (6.8%) women with hyperuricemia were treated with allopurinol (*p* < 0.01). There was an increase in the frequency of hyperuricemia with age, both in men and women (Figure 3) from 22.3% in men and 19.9% in women aged 65–69 years to 30.6% and 34.0% aged 90 years or above, respectively. When applying the same SUA cut-off (>6 mg/dL) for the definition of hyperuricemia in both sexes, the observed frequency would be much higher in men (N = 846; 40.3%) than in women (N = 544; 28.2%; *p* < 0.001).

### 3.2. Risk Factors of Hyperuricemia

Factors related to the risk of hyperuricemia were defined based on the logistic regression (Table 2). Factors that increased the risk of hyperuricemia were: obesity (including visceral obesity as well as being overweight), female gender, older age, diabetes, hypertension, CHF, CVD and hypertriglyceridemia, low eGFR, increased CRP, high personal income, and medication with aspirin and diuretics.

Results of the LASSO logistic regression are presented in Table 3. Ten risk factors of hyperuricemia, based on the ten-fold cross-validation with λ_opt_ = 59.2 (SE = 0.0168), were selected: obesity, diabetes, CHF, CVD, hypertriglyceridemia, eGFR < 60 mL/min/1.73 m^2^, CRP > 3.0 mg/dL, and medications such as hydrochlorothiazide, thiazide-like diuretics, and loop-diuretics. These factors were included in the multivariable logistic regression to show the strength of the association with hyperuricemia (Table 3). The analysis revealed that low glomerular filtration rate (eGFR < 60 mL/min/1.73 m^2^) and the use of loop diuretics were the strongest correlates of hyperuricemia (OR = 4.10 and 4.20, respectively) and more pronounced than the effect of hydrochlorothiazide and thiazide-like diuretics (OR = 2.96 and 2.81). Among components of the metabolic syndrome, hypertriglyceridemia (OR = 1.88) was the strongest correlate of hyperuricemia. From the analyzed factors, only diabetes appeared to not be significant.

### 3.3. The Use of Xanthine Oxidase Inhibitors

This analysis included 2519 men and 2354 women (mean age of both sexes was 79 ± 9 years). Allopurinol, as the only XOI and ULT included in the study, was used more often by men than women (N = 71; 2.8% vs. N = 42; 1.8%; *p* < 0.05) with a median dose of 100 mg daily. Only 29 (25.7%) participants were prescribed doses higher than 100 mg daily. There was no difference in allopurinol use between younger (65–79 years, N = 2507) and older (≥80 years, N = 2366) participants (2.2% vs. 2.4%; *p* = 0.68). In addition, five men used colchicine periodically in the therapy of gout.

In subjects treated with allopurinol, the mean SUA level was 6.2 ± 1.5 mg/dL (95% CI: 5.9–6.5) for the whole study group, and was significantly higher (*p* < 0.001) in men (6.6 ± 1.4 mg/dL; 95% CI: 6.2–7.0) than in women (5.6 ± 1.5 mg/dL; 95% CI: 5.1–6.1).

The therapy with allopurinol was considered effective (SUA < 6 mg/dL) in 45 (45.4%) of those treated, including 21 (46.7%) men and 24 (53.3%) women; *p* < 0.01. The lack of effectiveness was explained by the low (≤100 mg/day) prescribed dose of allopurinol in 75.0% (36 of 48) of men, and in 26.5% (13 of 32) of women. Higher doses (≥150 mg/day) were effective in all women and ineffective in 5 out of 14 men.

## 4. Discussion

Our study is the first presenting epidemiological data concerning the prevalence of hyperuricemia and accessing the prevalence of use of XO inhibitors in the older Polish population, including very long-lived individuals, which is unique. The most relevant risk factors contributing to the development of hyperuricemia were eGFR < 60 mL/min/1.73 m^2^, the use of diuretics (hydrochlorothiazide, thiazide-like and loop-diuretics), and the occurrence of obesity, heart failure, hypertriglyceridemia, and systemic inflammation.

Based on the obtained data, we estimated the population prevalence of hyperuricemia at 23.1% among older adults, and 25.5% in women and 19.2% in men. This makes the Polish prevalence one of the highest in older populations reported worldwide [27,38,39,40,41,42,43]. This is caused by the inclusion of a large percentage of octa- and nonagenarians, with a 1.48-times higher frequency of hyperuricemia in relation to younger seniors. The oldest were characterized by the highest rates of CKD, which is the leading risk factor for hyperuricemia and gout [51]. Subjects with eGFR < 30 mL/min/1.73 m^2^ have a 20-fold higher risk of hyperuricemia compared to the general population [30]. The prevalence of CKD in the older Polish adult population was estimated at 29.4%, with the frequency increasing from 17.5% in women and 18.5% in men aged 65–69 years to 66.5% and 64.8% in nonagenarians, respectively [52]. This is in line with kidney aging, manifested by an annual drop in GFR estimated at about 1 mL/min/m^2^ [53]. It seems that accelerated deterioration of the kidney excretory function in the oldest adults may be caused by low fluid consumption. Available data on the daily consumption of fluids acquired from the PolSenior study show that the minimal amount of fluids, defined as 1000–1500 mL daily, is consumed by 36.8% of the respondents and an insufficient amount of fluids, i.e., <1000 mL daily, by 11.4%. The number of people consuming insufficient amounts of fluids increases with age, from 7.4% in the 65–69 age group to 16.7% in the >90-year-old group, reaching the highest rates in nonagenarian women—20.3% [54].

Our data support the association between the use of diuretics and hyperuricemia. Diuretics are still one of the base medications in the treatment of hypertension, especially recommended in older adults [55]. In the PolSenior cohort, 29.7% of participants were receiving diuretics, mostly thiazide-like. The use of loop diuretics was the strongest risk factor for hyperuricemia, alongside eGFR < 60 mL/min/1.73 m^2^. The ORs for hydrochlorothiazide and thiazide-like diuretics were similar. Our observations are consistent with the results of the Atherosclerosis Risk in Communities Cohort (ARIC) study that investigated the effect of diuretic treatment on SUA concentration and the development of gout in 2169 adults with hypertension. It was shown that both loop and thiazide-like diuretics increased SUA, and are related to an increase in the incidence of gout. Furthermore, 330 ARIC patients who recently started the hypertension treatment with loop or thiazide-like diuretics had a higher mean SUA level by 0.96 mg/dL and 0.65 mg/dL, respectively, compared to those receiving non-diuretic treatment [34].

Obesity is an acknowledged risk factor for hyperuricemia occurrence [35,56,57]. Bad eating habits (unhealthy diet, high energy intake), low physical activity and obesity-related insulin-resistance play an important role in increasing SUA concentration [37]. The association between obesity and hyperuricemia is supported by the observed decrease in SUA levels in obese people losing weight due to dietary changes or bariatric therapy [57]. Consequently, weight reduction in overweight patients with high SUA levels and gout is recommended [32,57]. In the elderly Polish population, overweight and obesity were detected in 41.2% and 32.2%, respectively. Overweight, including obesity, was the second strongest component of metabolic syndrome (MetS), increasing the risk of hyperuricemia, following hypertriglyceridemia which was diagnosed in 25.8% of participants. Therefore, the association between hypertriglyceridemia and elevated SUA is well documented [32,56,58].

High cooccurrence of hyperuricemia, high serum triglyceride levels, and obesity may be explained by dietary habits in the older Polish population—high consumption of meat but low consumption of fluids and vegetables, as well as physical activity declining with age. Previously performed study showed that older adults eat too much animal fats such as butter, lard, hard cheeses, sausages, and meat, as well as sugar and other sweets, which also contribute to high SUA levels [59]. In the PolSenior cohort, a vegetarian diet was declared only by 1.4% of participants. Consumption of meat, fruit, and vegetables in European countries, among the population aged over 50, was assessed in the SHARE study in 16 European countries and Israel. It was found that, most often, meat was consumed three to six times a week, and 40% of people consumed meat every day. Additionally, the consumption of vegetables and fruits every day in the cited study was declared by 80% of respondents [60]. Representative data concerning the consumption of meat, vegetables, and fruit in older Polish adults are still not available.

Physical activity of middle-older age adults decreases with age. Half of the people aged over 65 years in Poland are physically inactive, and the decrease in recreational physical activity performed at least once a week decreases from 50.4% at the age of 65–69, to 15% at the age of 85–89, and 8.7% after the age of 90. The reasons for the reduced physical activity of the elderly are usually poor health (73.3%) and lack of feeling the need for recreation (30.4%) [54].

Among the factors associated with hyperuricemia were increased CRP levels. It was shown that increased CRP serum concentration is found in patients with MetS, with obesity being the major determinant of CRP levels [61,62,63]. This is explained by the production of inflammatory cytokines such as interleukin-6 (IL-6) in the adipose tissue [63]. Higher CRP values are also present in patients with increased SUA levels [61]. One reason is that obesity and MetS are the common cause for both disorders. Another is that excessive SUA leads to systemic inflammation by affecting the endothelium in the blood vessels and the deposition of monosodium urate crystals in soft tissues which, through macrophages and neutrophils, induces the production of pro-inflammatory cytokines such as interleukin-1β (IL-1β). We found elevated CRP values (>3 mg/dL) in 41.6% of participants which corresponds with the number of obese elders in the PolSenior study.

Another explanatory factor for hyperuricemia in our study was CHF. It was shown that SUA levels increased proportionally to the NT-proBNP concentration, and inversely to the left ventricular ejection fraction (LVEF), regardless of renal function impairment, BMI, or the use of thiazide diuretics [64]. Older adults account for around 80% of all patients with CHF [65]. The percentage of CHF in PolSenior participants (6.2%) was probably underestimated, because the diagnosis was based on the participants’ self-report. Additional studies should be performed to establish the mechanisms of CHF impact on SUA.

One of the milestones of cardiovascular prevention remains the use of low doses of acetylsalicylic acid. For prophylaxis with aspirin in patients with CVD and concomitant gout, the use of XOI should be considered, because the drug inhibits the excretion of UA with urine. This is explained by the *cis*-inhibition of URAT1 in the kidneys and the disruption of UA secretory action of organic anion transporters 1 and 3 (OAT1, OAT3) both contributing to hyperuricemia [66]. Low aspirin doses (≤325 mg daily), were shown to increase the risk of gout [67]. Of note, the use of acetylsalicylic acid was one of the weakest risk factors for high SUA concentrations in our study.

In addition to the analysis of the risk factors associated with the occurrence of hyperuricemia, we assessed the frequency of use and efficacy of ULT. Allopurinol was the only urate-lowering drug included in the analysis, because uricosurics (probenecid, benzbromarone) and febuxostat were not available in Poland and were not widely used at the time of the study. It should be stressed that few studies provide population data on the frequency of ULT. In the PolSenior population, XO inhibitors were used by only 2.3% of participants (9.3% of hyperuricemic participants), more frequently by men (2.8%) than women (1.8%). In addition, the used allopurinol doses were low, usually 100 mg daily. Only one-fourth of treated participants were prescribed with higher doses. Consequently, the therapy was only effective (SUA < 6 mg/dL) in 45.4% of them, more often in women (53.3%). It is estimated that in patients with gout, drugs lowering SUA are used correctly by approximately 50% (according to some data this is 10–46%), whereas non-compliance with treatment recommendations is more common in younger people [68,69]. This observation is consistent with other investigations, suggesting that less than 50% of patients on ULT achieve SUA levels < 6 mg/dL at an allopurinol dose of 300 mg/day [31]. Other studies have shown that 36% of participants treated with 100 mg and 300 mg of allopurinol required dose up-titrations to achieve an SUA level below 6 mg/dL [70]. The reason for the suboptimal management of hyperuricemia in older adults remains to be elucidated.

The main limitation of our study was the lack of data concerning the consumption of meat, dairy products, and vegetables, as well as incidences of gout. The PolSenior project was designed to cover the most important aspects of aging, and gout was not on the list. Nevertheless, we measured SUA in a large community-based cohort with high representation of the oldest members of society, which enabled us to study the correlates of hyperuricemia and analyze its frequency and management in such unique population.

## 5. Conclusions

Hyperuricemia was present in 23.1% of the older Polish population. The frequency of hyperuricemia increases with age, reaching 30.5% in men and 33.7% in women aged 90 years or more. Chronic kidney disease and the use of diuretics are the strongest risk factors for hyperuricemia in the older Polish population, followed by hypertriglyceridemia, obesity, and heart failure. XO inhibitors were used by 2.3% of participants and the efficiency of ULT was 45.4%, mainly due to low doses of allopurinol.

## Figures and Tables

**Table 1:** Characteristics of the study group included in the analysis of hyperuricemia for all analyzed subjects (N = 4028), and for men and women separately.

	All	Men N = 2101 (52.2%)	Women N = 1927 (47.8%)
Age (years)	79 ± 9	79 ± 8	79 ± 9
65–69 years, N (%)	683 (16.9)	327 (15.6)	356 (18.5)
70–74 years, N (%)	778 (19.3)	399 (19.0)	379 (19.7)
75–79 years, N (%)	696 (17.3)	372 (17.7)	324 (16.8)
80–84 years, N (%)	627 (15.6)	338 (16.1)	289 (15.0)
85–89 years, N (%)	684 (17.0)	387 (18.4)	297 (15.4)
≥90 years, N (%)	560 (13.9)	278 (13.2)	282 (14.6)
Rural area residence, N (%)	2395 (59.4)	1254 (59.7)	1141 (59.2)
Blue-collar, N (%)	1365 (33.9)	821 (39.1)	544 (28.2)
White-collar, N (%)	326 (8.1)	216 (10.4)	110 (5.8)
Living alone, N (%)	816 (20.2)	279 (13.4)	537 (28.2)
Personal income			
• Low, N (%)	1433 (40.5)	491 (26.9)	942 (55.1)
• Average, N (%)	1753 (49.6)	1042 (57.0)	711 (41.6)
• High, N (%)	351 (9.9)	294 (16.1)	57 (3.3)
Vegetarian diet, N (%)	57 (1.4)	31 (1.5)	26 (1.3)
Alcohol consumers “drinking”, N (%)	628 (15.6)	520 (25.1)	108(5.7)
BMI (kg/m^2^)	28.1 ± 5.0	27.4 ± 4.4	29.0 ± 5.5
Overweight, N (%)	1570 (41.2)	912 (45.3)	658 (36.5)
Obesity, N (%)	1230 (32.2)	512 (25.4)	718 (39.8)
Visceral obesity, N (%)	3158 (81.2)	1505 (73.4)	1653 (89.8)
Diabetes, N (%)	925 (23.0)	447 (21.3)	478 (24.8)
Hypertension, N (%)	2945 (73.4)	1446 (69.1)	1499 (78.1)
Coronary artery disease, N (%)	865 (21.5)	492 (23.4)	373 (19.4)
Heart failure, N (%)	243 (6.2)	140 (6.8)	103 (5.5)
Hypercholesterolemia, N (%)	2973 (73.8)	1408 (67.0)	1565 (81.2)
Hypertriglyceridemia, N (%)	1038 (25.8)	446 (21.2)	592 (30.7)
eGFR (mL/min/1.73m^2^)	65.7 ± 18.4	67.5 ± 18.7	63.6 ± 17.8
45–59.9 mL/min/1.73m^2^, N (%)	976 (24.2)	466 (22.2)	510 (26.5)
30–44.9 mL/min/1.73m^2^, N (%)	397 (9.9)	187 (8.9)	210 (10.9)
<30 mL/min/1.73m^2^, N (%)	97 (2.4)	39 (1.9)	58 (3.0)
hs-CRP (mg/dL)	2.36 (1.12–4.94)	2.28 (1.02–5.01)	2.44 (1.21–4.86)
>3 mg/dL, N (%)	1664 (41.6)	854 (40.9)	810 (42.3)
Hydrochlorothiazide, N (%)	243 (6.0)	109 (5.2)	134 (7.0)
Thiazide-like, N (%)	623 (15.5)	257 (12.2)	366 (20.0)
Loop diuretics, N (%)	387 (9.6)	202 (9.6)	185 (9.6)
Spironolactone, N (%)	469 (11.6)	226 (10.8)	243 (12.6)
Aspirin, N (%)	1367 (33.9)	725 (34.5)	642 (33.3)

mean ± standard deviation or median (lower–upper quartile).

**Table 2:** Risk factors of hyperuricemia in univariable logistic regression.

Variable	β	SE (β)	OR	±95% CI	*p*
Female gender	0.1789	0.0715	1.196	1.039–1.376	<0.05
Age (per 5 years)	0.1205	0.0041	1.128	1.119–1.137	<0.001
Age ≥80 years	0.3886	0.0717	1.475	1.281–1.698	<0.001
Rural area residence	0.1614	0.0734	1.175	1.017–1.357	<0.05
White-collar	−0.1417	0.1351	0.868	0.666–1.131	0.29
Living alone	0.0416	0.0885	1.047	0.880–1.246	0.60
Income—average vs. low	0.1409	0.0815	1.151	0.981–1.351	0.08
Income–high vs. low	0.3509	0.1302	1.420	1.100–1.833	<0.01
Vegetarian diet	−0.3031	0.3269	0.738	0.389–1.402	0.35
Alcohol consumption	−0.0093	0.0988	0.991	0.816–1.202	0.92
BMI (kg/m^2^)	0.0900	0.0075	1.094	1.078–1.110	<0.001
Overweight vs. normal weight	0.4328	0.1027	1.542	1.261–1.885	<0.001
Obesity vs. normal weight	1.0488	0.1026	2.854	2.334–3.490	<0.001
Visceral obesity	0.6444	0.1056	1.905	1.549–2.343	<0.001
Diabetes	0.5368	0.0808	1.710	1.460–2.004	<0.001
Hypertension	0.4972	0.0870	1.644	1.386–1.950	<0.001
Coronary artery disease	0.6130	0.0821	1.846	1.571–2.168	<0.001
Heart failure	0.9940	0.1340	2.702	2.078–3.514	<0.001
Hypercholesterolemia	0.0177	0.0814	1.018	0.868–1.194	0.83
Hypertriglyceridemia	0.7510	0.0775	2.118	1.820–2.466	<0.001
eGFR (per 10 mL/min/1.73 m^2^)	−0.6780	0.0028	0.508	0.505–0.510	<0.001
eGFR 45–59.9 vs. > 60 mL/min/1.73 m^2^	1.1669	0.0879	3.212	2.704–3.816	<0.001
eGFR 30–44.9 mL/min/1.73 m^2^	2.5986	0.1224	13.446	10.577–17.092	<0.001
eGFR < 30 mL/min/1.73 m^2^	3.6636	0.3034	39.001	21.520–70.682	<0.001
hs-CRP (mg/dL)	0.0222	0.0041	1.022	1.014–1.031	<0.001
hs-CRP > 3 mg/dL vs	0.6351	0.0725	1.887	1.637–2.176	<0.001
Medication:					
Hydrochlorothiazide	1.0431	0.1325	2.838	2.189–3.679	<0.001
Thiazide-like	0.8853	0.0888	2.424	2.036–2.885	<0.001
Loop diuretics	1.7489	0.1113	5.748	4.621–7.150	<0.001
Spironolactone	1.0010	0.0988	2.721	2.242–3.302	<0.001
Aspirin	0.3012	0.0741	1.351	1.169–1.563	<0.001

**Table 3:** Risk factors of hyperuricemia. The results of LASSO and multivariable logistic regression.

Variable	LASSO	OR	±95% CI	*p*
Obesity vs. normal weight	0.2833	1.746	1.455–2.094	<0.001
Diabetes	0.0141	1.163	0.954–1.418	0.13
Coronary artery disease	0.0185	1.303	1.068–1.589	<0.05
Heart failure	0.1937	1.700	1.380–1.945	<0.001
Hypertriglyceridemia	0.4191	1.884	1.565–2.268	<0.001
eGFR < 60 mL/min/1.73 m^2^	1.2297	4.096	3.445–4.869	<0.001
hs-CRP > 3 mg/dL	0.2853	1.638	1.380–1.945	<0.001
Hydrochlorothiazide	0.5706	2.965	2.181–4.030	<0.001
Thiazide-like diuretics	0.7260	2.809	2.287–3.451	<0.001
Loop diuretics	1.0688	4.203	3.213–5.496	<0.001

## Data Availability

The data presented in this study are available on request from the corresponding author.
